# Acute type A aortic dissection in patients with non-prior cardiac surgery vs. prior cardiac surgery: a systematic review and meta-analysis

**DOI:** 10.3389/fcvm.2024.1438556

**Published:** 2024-08-26

**Authors:** Muhammad Ahmed, Hafsah Alim Ur Rahman, Muhammad Ahmed Ali Fahim, Zahabia Altaf Hussain, Nisar Ahmed, Muhammad Sohaib Asghar

**Affiliations:** ^1^ Shaheed Mohtarma Benazir Bhutto Medical College, Lyari, Karachi, Pakistan; ^2^ Dow Medical College, Dow University of Health Sciences, Karachi, Pakistan; ^3^ Department of Internal Medicine, Rapides Regional Medical Center, Alexandria, LA, United States; ^4^ Department of Internal Medicine, AdventHealth, Orlando, FL, United States

**Keywords:** aortic dissection, cardiac surgery, meta-analysis, systematic literature search, PRISMA

## Abstract

**Background:**

Patients with prior cardiac surgery undergoing acute type A aortic dissection (ATAAD) are thought to have worse clinical outcomes as compared to the patients without prior cardiac surgery.

**Aim:**

To compare the safety and efficacy of ATAAD in patients with prior cardiac surgery.

**Methods:**

We systematically searched PubMed, Cochrane Library and Google Scholar from database inception until April 2024. We included nine studies which consisted of a population of 524 in the prior surgery group and 5,249 in the non-prior surgery group. Our primary outcome was mortality. Secondary outcomes included reoperation for bleeding, myocardial infarction, stroke, renal failure, sternal wound infection, cardiopulmonary bypass (CPB) time, cross-clamp time, hospital stay, and ICU stay.

**Results:**

Our pooled estimate shows a significantly lower rate of mortality in the non-prior cardiac surgery group compared to the prior cardiac surgery group (RR = 0.60, 95% CI = 0.48–0.74). Among the secondary outcomes, the rate of reoperation for bleeding was significantly lower in the non-prior cardiac surgery group (RR = 0.66, 95% CI = 0.50–0.88). Additionally, the non-prior cardiac surgery group had significantly shorter CPB time (MD = −31.06, 95% CI = −52.20 to −9.93) and cross-clamp time (MD = −21.95, 95% CI = −42.65 to −1.24). All other secondary outcomes were statistically insignificant.

**Conclusion:**

Patients with prior cardiac surgery have a higher mortality rate as compared to patients who have not undergone cardiac surgery previously. Patients with prior cardiac surgery have higher mortality and longer CPB and cross-clamp times. Tailored strategies are needed to improve outcomes in this high-risk group.

## Introduction

Aortic dissection is a life-threatening condition caused by a tear in the inner layer of the aorta or bleeding within the aortic wall, resulting in the separation of its layers. It commonly occurs in individuals aged 65–75 (1). Risk factors include older age, male gender, hypertension, and aortic aneurysms. Genetic conditions like Marfan syndrome, Loeys-Dietz syndrome, Ehlers-Danlos syndrome, and bicuspid aortic valves increase the risk in younger individuals (2). Diagnosing aortic dissection can be challenging as not all cases present with the classic symptom of sudden, severe chest pain that spreads to the back. Symptoms may include abdominal pain, flu-like symptoms, vomiting, diarrhea, lower back pain, stroke-like symptoms, and fainting (3). The Stanford system categorizes aortic dissections into two types based on the involvement of the ascending or descending aorta. Type A involves the ascending aorta, while Type B originates in the descending aorta past the left subclavian artery (4). Type A aortic dissection poses a more imminent risk to life compared to Type B. Type A is more life-threatening, with complications such as pericardial tamponade, rupture, aortic valve dysfunction, or coronary artery malperfusion (5). Stroke, a concerning complication of acute type A aortic dissection (ATAAD), occurs in over 5% of patients and is linked to increased morbidity and in-hospital mortality. This is primarily due to brain tissue ischemia from hypotension and compromised cerebral circulation, with neurological symptoms appearing in 17%–40% of cases. Predominant manifestations include ischemic stroke in 6%–32% of cases, notably right hemispheric, and occasionally bilateral strokes (6, 7). Autopsy findings show aortic dissection prevalence of 1%–3%, with an annual incidence of Type A at 3 per 100,000 people (8). Immediate surgical intervention is crucial for acute Type A cases. Despite advancements in diagnosis, initial care, and clinical awareness, surgical repair survival rates remain low, with an in-hospital mortality rate of 16%–18% (9).

Acute aortic dissection occurs in 0.12%–0.16% of patients with prior cardiac surgery and 0.6% of those with previous aortic valve replacement. Approximately 1 in 7–8 ATAAD patients have had prior cardiac surgery (10). Primary ATAAD requires rapid diagnosis and surgery due to a 50% mortality rate within 48 h. However, Type A dissection following previous cardiac surgery presents unique challenges and should be considered a distinct category. These patients typically appear hemodynamically stable, with rare occurrences of cardiac tamponade and free rupture (11). Additionally, previous cardiac surgeries are linked to higher postoperative bleeding and worse outcomes in subsequent elective procedures. ATAAD in these patients is particularly challenging due to chest reentry, potential graft damage, mediastinal structure isolation, and prolonged surgical times, which complicate myocardial and cerebral protection (12).

Several studies have systematically reviewed and analyzed outcomes in acute Type A aortic dissection (13, 14). However, the outcomes of Type A aortic dissection in patients with prior cardiac surgery compared to those without prior cardiac surgery have not been investigated yet. A comprehensive analysis of outcomes following surgical repair for Type A aortic dissection found that prior cardiac surgery was identified as an independent risk factor (9). Given the critical nature of these findings, it is essential for clinicians to understand the nuances of managing patients with a history of cardiac surgery who develop ATAAD. Such knowledge can significantly impact preoperative assessment, surgical strategy, and postoperative care, ultimately influencing patient prognosis and survival rates.

After an extensive literature search, we found no existing meta-analyses in this regard. Therefore, our systematic review and meta-analysis aim to predict and compare outcomes of acute type A aortic dissections in patients with and without prior cardiac surgery.

## Methods

This meta-analysis and systematic review was conducted according to the established guidelines by Cochrane and Preferred Reporting Items for Systematic Review and Meta-Analysis (PRISMA) (15). Since we used data from already published literature and did not collect any new patient data, this study did not require approval from the institutional review board.

### Literature search strategy

The research team conducted a systematic search for the published literature on multiple databases which included PubMed, Google Scholar, and Cochrane Library. We searched for studies published from inception till April 2024 using the keywords “acute type A aortic dissection”, “prior cardiac surgery”, “non-prior cardiac surgery”. Moreover, we also identified articles from the reference lists of the relevant studies to be included in our library of studies. A detailed search string containing all the pertinent keywords used during the search is outlined in Supplementary Table S1.

### Study selection and eligibility criteria

All the articles retrieved from the search were imported to EndNote X9 Reference Manager (Clarivate Analytics, Philadelphia, Pennsylvania) and duplicates were removed. The remaining articles were screened for relevancy through title and abstract by two independent researchers (M.A and H.A.U.R). Full texts of the shortlisted articles were assessed for the presence of relevant intervention and control groups, outcomes of interest, methodology. Disagreements were resolved with the consensus of the third author (M.A.A.F). We shortlisted nine studies which directly compared the outcomes of prior cardiac surgery with non-prior cardiac surgery in patients with acute type A aortic dissection. We included studies which presented data of interest and studies that did not have comparative groups and data that could not be analyzed were excluded.

### Data extraction

Two authors (M.A and H.A.U.R) independently extracted data from the shortlisted studies on an excel sheet. Important data pertinent to the trial (author name, year) and participants at baseline (sample size, age), baseline characteristics were collected. Primary and secondary outcomes were also recorded in the excel sheet which included mortality, reoperation for bleeding, myocardial infarction, stroke, renal failure, sternal wound infection, cardiopulmonary bypass (CPB) time, cross-clamp time, hospital stay, and ICU stay. Mortality was chosen as the primary outcome because it is the key measure of surgical success and prognosis in acute type A aortic dissection. This focus allows clear comparison of surgery effectiveness between patients with and without prior cardiac surgery.

### Risk of bias and quality assessment

Quality assessment for the included observational studies which was done using Newcastle Ottawa Scale (NOS). All the included studies had low risk of bias across the three domains of selection, comparability, and outcome. Detailed quality assessment is provided in the Supplementary Table S2.

### Statistical analysis

We used Review Manager (V.5.4.1 Cochrane Collaboration, London, United Kingdom) to perform the statistical analysis. Risk ratios (RR) were calculated for dichotomous outcomes and mean differences (MD) were calculated for continuous outcomes with 95% confidence intervals (CI). A random effects model was used to evaluate all the outcomes. The heterogeneity across pooled studies was assessed using Higgins *I*^2^ statistics. A value of *I*^2^ = 25%–50% was considered mild, 50%–75% as moderate, and greater than 75% as severe heterogeneity (16). To justify heterogeneity, we also performed sensitivity analysis for the outcomes which had severe heterogeneity. The *p*-value of <0.05 was considered significant throughout our analysis.

## Results

### Study selection and characteristics

A comprehensive literature search was conducted has yielded 5,051 articles. Upon removing duplicates and removing ineligible articles, nine studies were included in this meta-analysis. The PRISMA flowchart presents the summary of the literature search in Supplementary Figure S1. A total of nine studies comprising 5,763 patients (5,249 in non-prior cardiac surgery (CS) group vs. 524 in prior cardiac surgery (PCS) group). The mean age of patients in the non-prior cardiac surgery group was 57.82 years, and 60.12 years in the prior cardiac surgery group. A detailed characteristics of the included studies and patients is summarized in Table 1.

**Table 1:** General and baseline characteristics of the included studies.

Author, year	Patient population		Average age (years), mean (SD) or median (IQR)		Male sex, *n* (%)		Diabetes mellitus, *n* (%)		Hypertension, *n* (%)		Coronary artery disease, *n* (%)		Chronic kidney disease, *n* (%)		Chronic obstructive pulmonary disease, *n* (%)		Connective tissue disorder, *n* (%)		Any malperfusion, *n* (%)		Prior stroke, *n* (%)	
	Non-prior cardiac surgery	Prior cardiac surgery	Non-prior cardiac surgery	Prior cardiac surgery	Non-prior cardiac surgery	Prior cardiac surgery	Non-prior cardiac surgery	Prior cardiac surgery	Non-prior cardiac surgery	Prior cardiac surgery	Non-prior cardiac surgery	Prior cardiac surgery	Non-prior cardiac surgery	Prior cardiac surgery	Non-prior cardiac surgery	Prior cardiac surgery	Non-prior cardiac surgery	Prior cardiac surgery	Non-prior cardiac surgery	Prior cardiac surgery	Non-prior cardiac surgery	Prior cardiac surgery
Bjurbom et al. (20)	1,119	40	61.4 ± 12.2	65.0 ± 10.6	753 (67.3)	31 (77.5)	25 (2.2)	1 (2.5)	574 (51.3)	25 (62.5)	42 (3.8)	18 (45)	19 (1.7)	2 (5)	68 (6.1)	1 (2.5)	–	–	367 (32.8)	14 (35)	46 (4.1)	1 (2.5)
Brown et al. (21)	529	72	61.4 ± 13.4	61.2 ± 13.6	311 (58.8)	50 (69.4)	44 (8.3)	19 (26.4)	392 (74.1)	65 (90.3)	58 (11.0)	28 (38.9)	–	–	71 (13.4)	15 (20.8)	–	–	173 (32.7)	17 (23.6)	–	–
D'Onofrio et al. (12)	1,387	85	66 (56–75)	67 (56–74)	585 (42)	40 (47)	69 (5.4)	2 (2.5)	907 (71)	59 (75)	92 (7.5)	18 (22)	102 (7.9)	9 (11)	95 (7.5)	7 (9.0)	13 (1.1)	3 (3.8)	310 (34)	18 (32)	42 (3.5)	9 (12)
Estrera et al. (17)	281	59	57.9 ± 13.9	63 ± 12.8	187 (67)	40 (82)	–	–	–	–	–	–	–	–	–	–	–	–	–	–	29 (10)	1 (2)
Ge et al. (22)	32	32	38.97 ± 13.14	45.13 ± 12.04	27 (84.64)	28 (87.5)	–	–	17 (53.13)	16 (50.00)	–	–	–	–	–	–	11 (34.38)	10 (31.25)	–	–	–	–
Klodell et al. (23)	31	31	64.3 ± 13.1	66.9 ± 14.4	29 (93.5	28 (90.3)	1 (3.2)	3 (9.7)	27 (87.1)	25 (80.6	–	–	5 (16.1)	6 (19.4)	7 (22.6)	7 (22.6)	–	–	–	–	–	–
Krebs et al. (18)	1,194	138	58 (48–69)	63 (53–71)	786 (65.8)	104 (75.4)	143 (12.0)	32 (23.2)	964 (80.7)	124 (89.9)	–	–	–	–	49 (4.1)	15 (10.9)	–	–	–	–	–	–
Modi et al. (24)	103	11	63 ± 12.3	59.5 ± 14.7	65 (63.1)	9 (81.8)	–	–	–	–	–	–	–	–	–	–	4 (3.9)	2 (18.2)	–	–	–	–
Rylski et al. (19)	573	56	60 (50–72)	70 (60–75)	365 (64)	38 (68)	50 (9)	8 (14)	450 (79)	50 (89)	73 (13)	38 (68)	45 (8)	5 (9)	53 (9)	6 (11)	29 (5)	0	–	–	41 (7)	5 (9)

### Primary outcome

**Figure 1:**
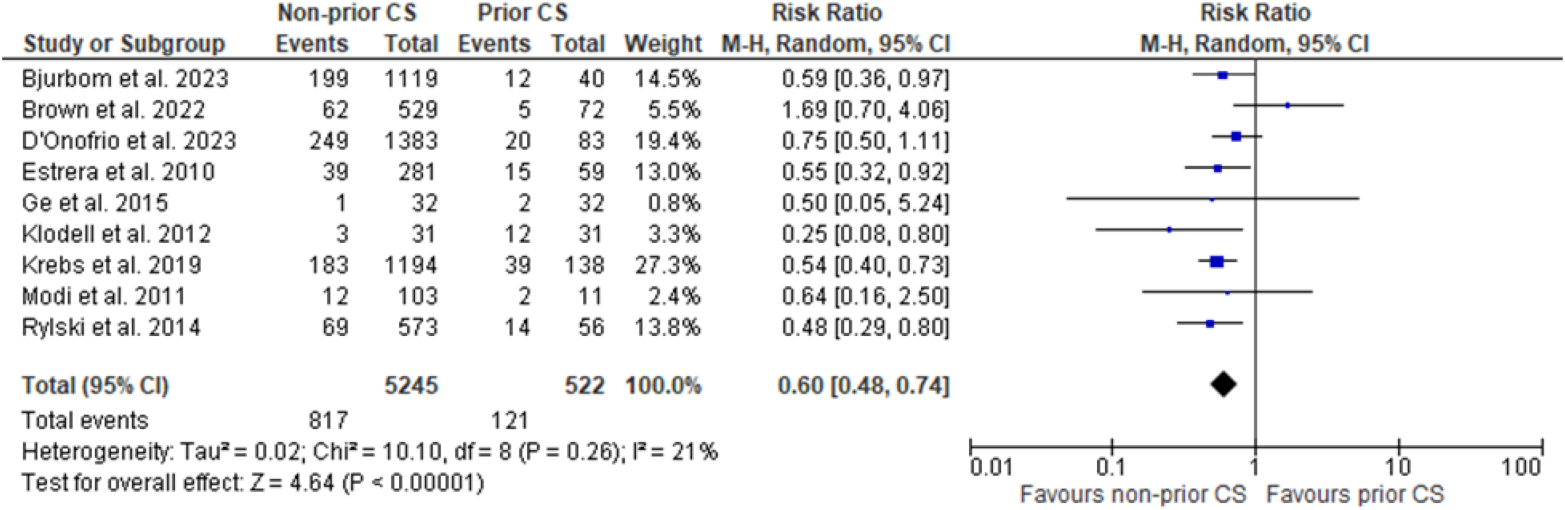
Forest plot of mortality.

Across nine studies our primary outcome of mortality was assessed, and pooling data resulted in a decreased occurrence in patients receiving no prior cardiac surgery as compared to those with prior cardiac surgery. Furthermore, this result was of statistical significance (RR = 0.60, 95% CI = 0.48–0.74; *p* = 0 < 0.00001; *I*^2^ = 21%). Forest plot for the outcome of mortality is shown in Figure 1.

**Figure 2:**
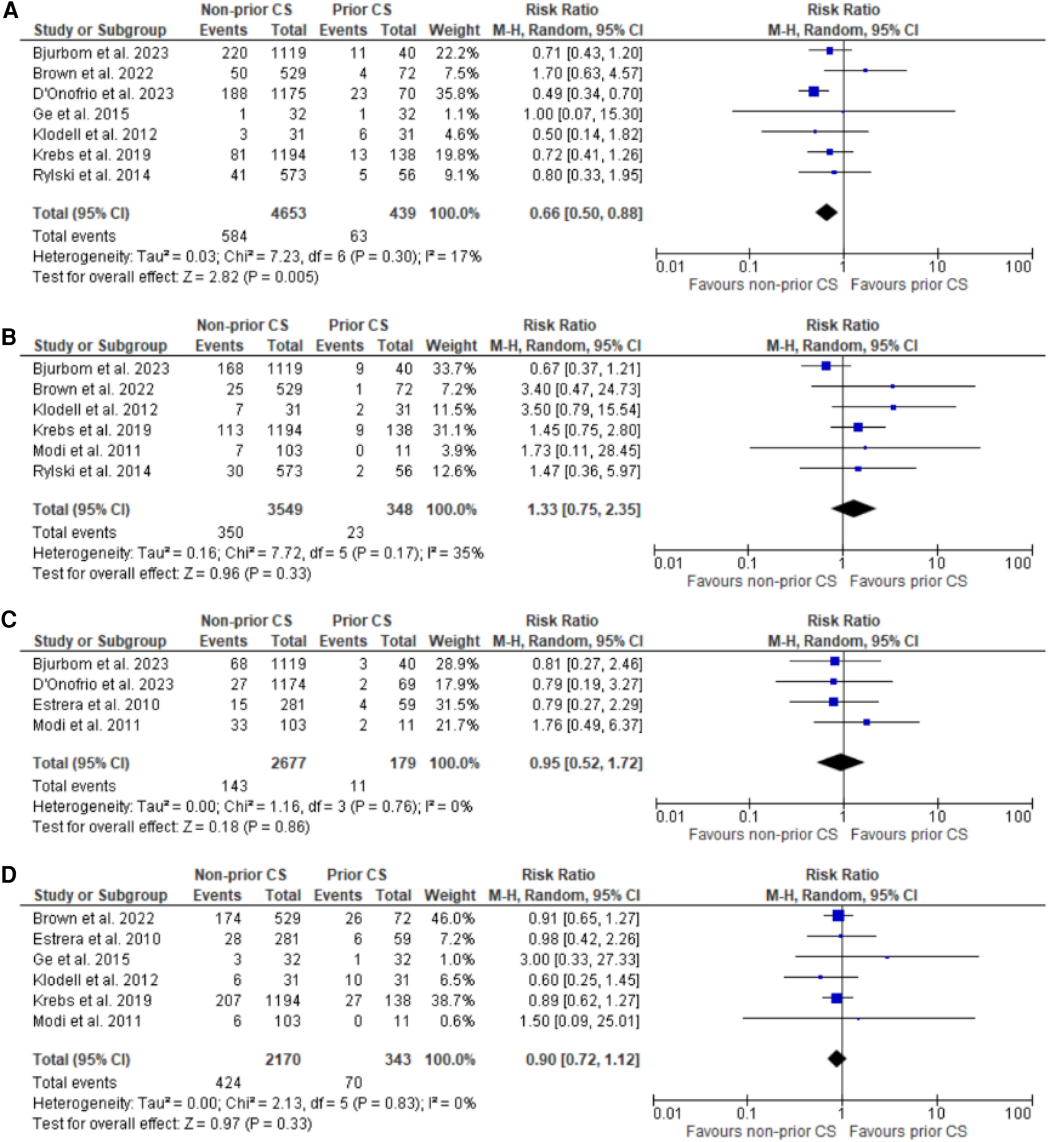
Forest plots for reoperation for bleeding, stroke, myocardial infarction, and renal failure. **(A)** Forest plot of reoperation for bleeding. **(B)** Forest plot of stroke. **(C)** Forest plot of myocardial infarction. **(D)** Forest plot of renal failure.

**Figure 3:**
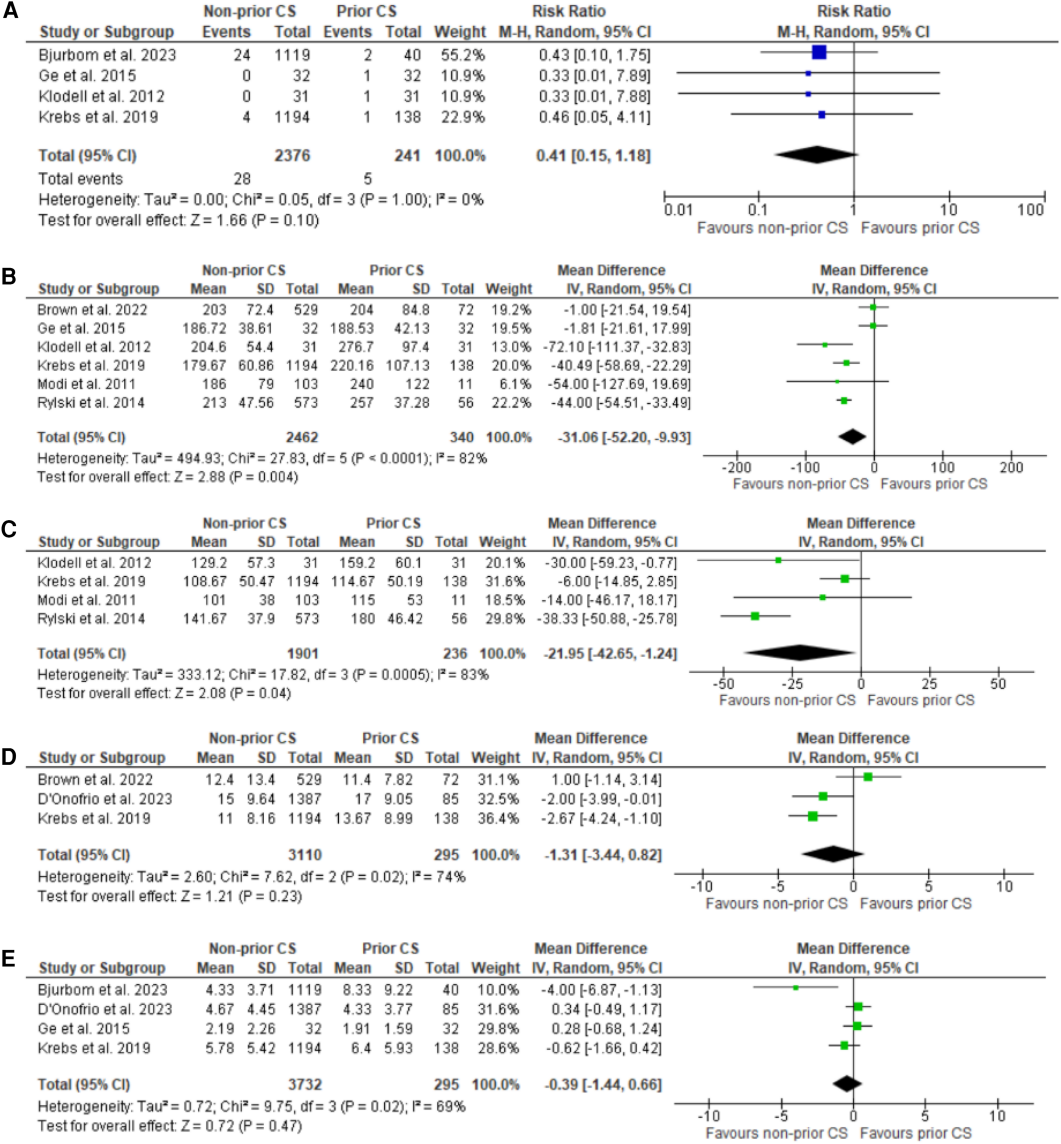
Forest plots for sternal wound infection, CPB time (minutes), cross clamp time (minutes), hospital and ICU stay (days). **(A)** Forest plot of sternal wound infection. **(B)** Forest plot for CPB time. **(C)** Forest plot of cross-clamp time. **(D)** Forest plot of hospital stay. **(E)** Forest plot of ICU stay.

### Secondary outcomes

The results for all the secondary outcomes are summarized in the tabular form in Table 2. Forest plots for the secondary outcomes are presented in Figures 2, 3.

**Table 2:** Results for the secondary outcomes.

Outcome	Studies	Effect-estimate (95% CI)	*p*-value	*I*^2^ (%)	Figures
Reoperation for bleeding	7	RR: 0.66 (0.50 to 0.88)	0.005	17	2A
Stroke	6	RR: 1.33 (0.75 to 2.35)	0.33	35	2B
Myocardial infarction	4	RR: 0.95 (0.52 to 1.72)	0.86	0	2C
Renal failure	4	RR: 0.90 (0.72 to 1.12)	0.33	0	2D
Sternal wound infection	4	RR: 0.41 (0.15 to 1.18)	0.10	0	3A
CPB (minutes)	7	MD: −31.06 (−52.20 to −9.93)	0.004	82	3B
Cross-clamp time (minutes)	4	MD: −21.95 (−42.65 to −1.24)	0.04	83	3C
Hospital stay (days)	3	MD: −1.31 (−3.44 to 0.82)	0.23	74	3D
ICU stay (days)	4	MD: −0.39 (−1.44 to 0.66)	0.47	69	3E

CPB, cardiopulmonary bypass time; CI, confidence interval; *I*^2^, heterogeneity; ICU, intensive care unit; MD, mean difference; RR, risk ratio.

### Sensitivity analysis

For initial analyses which revealed a significant (<75%) heterogeneity a leave-one-out analysis was performed sequentially omitting one study until a significantly lower heterogeneity was revealed. The exclusion of Krebs et al. (18) revealed a substantial difference in heterogeneity for the endpoint of cross clamp time (*I*^2^ = 83% to *I*^2^ = 0%) as shown in Supplementary Figure S2. For the end point of CPB time multiple outlier studies were identified based on modality of assessment of CPB and removed from the initial analysis producing a final dataset with no heterogeneity. (*I*^2^ = 82% to *I*^2^ = 0%) as shown in Supplementary Figure S3.

### Quality assessment

All of the included studies were found to have low risk of bias upon quality assessment by the NOS. All studies obtained a score of ≥8 which indicated that all the studies are of high quality as shown in the Supplementary Table S2.

## Discussion

In this meta-analysis, while comparing the outcomes of ATAAD in patients with and without prior cardiac surgery, we found a statistically significant reduction in mortality in individuals who had not had any prior surgery. ATAAD patients without prior surgery also showed a significantly lower risk of bleeding. Other studies have also found a greater mortality rate in ATAAD patients who had previously undergone cardiac surgery (25, 26).

It has been hypothesized that an increased risk of mortality in patients with prior cardiac surgery may be due to persistent aortic wall abnormalities, untreated or recurrent dissection, and injuries caused by aortic cross-clamp, intimal tears at the site of suture lines for bypass grafts, or cannulation during a prior cardiac surgery, all of which contribute to an increased risk of subsequent ascending aortic tear in such patients and hence, increased risk of adversities with it (25, 27). Additionally, postoperative complications such as coronary malperfusion and cardiac tamponade, as identified by Rylski et al., can further exacerbate the risk in patients with previous surgery (19). Other studies, however, have failed to show any such relation between mortality and prior cardiac surgery in ATAAD patients (28). Another significant issue is the presence of adhesions from previous surgeries, which can complicate subsequent procedures and increase the risk of intraoperative bleeding and other complications (29). Postoperative dissection challenges including residual or recurrent dissection, anastomotic leaks, and the formation of new entrance tears, can also lead to further aortic complications and increased mortality (30). This significant reduction in mortality found in individuals without prior surgery underscores the critical need for heightened awareness among surgeons regarding the increased risks faced by ATAAD patients with previous cardiac surgery. Tailored treatment strategies are essential to mitigate these risks. These strategies could include enhanced preoperative diagnostics such as detailed imaging studies to assess the integrity of the aortic wall, and more frequent monitoring for early signs of dissection or other complications (31). Postoperatively, these patients may benefit from closer surveillance with regular imaging and clinical evaluations to promptly identify and manage any complications or recurrent dissections (32).

Our study did not show any significant reduction in stroke, myocardial infarction, renal failure, or wound infection in ATAAD patients with prior cardiac surgery. This was contrary to the findings shown in a study conducted by Estrera et al. which showed a four times increased risk of stroke in patients with prior cardiac surgery (17). CPB and cross-clamp time were, however, decreased in patients with no prior cardiac surgery. This can be explained by the increased bleeding time in patients with a prior surgery, resulting in difficulty in cardiac protection and increased requirement of transfusion (17). Evaluating the observed disparities in outcomes requires considering the intricacies of CPB procedures. CPB is an important part of cardiac surgery because it provides critical circulatory and respiratory support during difficult procedures (33). Variations in CPB procedures among surgical institutions and research may contribute to disparities in results found between individuals with and without prior cardiac surgery. The efficiency and safety of CPB are influenced by factors such as the duration of bypass and cross-clamp time, cannulation site selection, and techniques for reducing ischemia-reperfusion harm (34). Understanding the influence of CPB procedures on outcomes in ATAAD patients may further provide valuable context and emphasizes the importance of standardized perioperative treatment in this high-risk population.

In addition to these results, our study failed to show any significant difference in hospital and ICU stay in both groups. Although some studies have shown an increased time spent by prior CS patients in the hospital, cost of stay was found to be same in both groups (18). Tailoring treatment options based on prior surgical history is a critical step toward providing tailored and effective care to patients with acute type A aortic dissection. Patients with a history of previous cardiac surgery face particular problems and considerations, necessitating specialist management strategies. Enhanced surveillance methods, including more regular monitoring and imaging investigations, may be necessary to detect and treat any problems or recurrent dissections in this high-risk category. Healthcare professionals can optimize outcomes and improve overall patient care in the management of ATAAD by tailoring treatment approaches to the specific needs and risks of patients who have previously undergone cardiovascular surgery.

The results of our meta-analysis should be interpreted in the light of certain limitations. The primary limitation of this study is related to the retrospective, non-randomized nature of the underlying studies. Despite efforts to eliminate bias, several biases inherent to retrospective cohort studies persist. Individuals are chosen after the event has occurred in these studies, which increases the possibility of selection bias. Furthermore, the retrospective comparative studies did not include long-term follow-up. Secondly, the meta-analysis was conducted under the assumption that the baseline characteristics of the participants in the included studies were substantially similar. Multiple confounding factors were not measured or adjusted in the results due to the absence of relevant details from the original studies. Unacknowledged or inadequately assessed confounders may undermine the link being inferred. As with other meta-analyses of observational studies, the data cannot be utilized for the determination of causal effects. Increased age in patients with prior cardiac surgery can be subject to confounding bias. Moreover, the heterogeneity in expertise levels of the surgeons performing the procedures may influence outcomes, adding another layer of bias. Furthermore, the inclusion of various types of procedures in the analyzed articles may have increased heterogeneity and potential bias. Due to the inability to perform subgroup analyses, all cardiac procedures were treated uniformly. Lastly, only English-language articles that had been published were included, which may introduce language bias in our study.

## Conclusion

In the light of the above analysis, we found an increased risk of mortality, increased bleeding and increased coronary bypass and cross-clamp time in minute in ATAAD patients with prior cardiac surgery. The present study suggests that a history of prior cardiac surgery should be considered while treating ATAAD patients to devise a patient-centered strategy.

## Data Availability

The original contributions presented in the study are included in the article/Supplementary Material, further inquiries can be directed to the corresponding author.
