# Supplementary material for: Acute type A aortic dissection in patients with non-prior cardiac surgery vs. prior cardiac surgery: a systematic review and meta-analysis
Source: Front Cardiovasc Med. 2024 Aug 26;11:1438556. doi: 10.3389/fcvm.2024.1438556 (PMC11381269; doi:10.3389/fcvm.2024.1438556)
Supplement: Supplementary file 1 [file Datasheet1.docx]

**Supplementary Material**

Supplementary Figure 1 – PRISMA flow chart

Supplementary Table 1 – Detailed search strategy used in each database.

Supplementary Table 2 – Newcastle-Ottawa Quality Assessment for cohorts

Supplementary Figure 2 – Leave-one-out analysis of cross-clamp time

Supplementary Figure 2 – Removing outlier studies in the analysis of CPB time

**Supplementary Figure 1 (S1).** PRISMA flowchart

**Identification of studies via databases and registers**

Records removed *before screening*:

Duplicate records removed
(n = 789)

Records identified from*:

PubMed (n =1079)

Google Scholar (n =3970)
Cochrane Library (n=2)

**Identification**

Records screened

(n =4262)

Records excluded**

(n = 2600)

Reports sought for retrieval

(n =1662)

Reports not retrieved

(n =120)

**Screening**

Reports assessed for eligibility

(n =1542)

Reports excluded:

Non-comparative group (n =679)

Outcome of interest not reported (n =854)

Reports of included studies

(n =9)

**Included**

*Consider, if feasible to do so, reporting the number of records identified from each database or register searched (rather than the total number across all databases/registers).

**If automation tools were used, indicate how many records were excluded by a human and how many were excluded by automation tools.

*From:*  Page MJ, McKenzie JE, Bossuyt PM, Boutron I, Hoffmann TC, Mulrow CD, et al. The PRISMA 2020 statement: an updated guideline for reporting systematic reviews. BMJ 2021;372:n71. doi: 10.1136/bmj.n71

For more information, visit: <http://www.prisma-statement.org/>

**Supplementary table 1.** Search strings used in each database.

| **PubMed** | ("Type A aortic dissection"[All Fields] OR "Acute type A aortic dissection"[All Fields] OR "ATAAD"[All Fields]) AND ((("prior"[All Fields] OR "priors"[All Fields]) AND ("thoracic surgery"[MeSH Terms] OR ("thoracic"[All Fields] AND "surgery"[All Fields]) OR "thoracic surgery"[All Fields] OR ("cardiac"[All Fields] AND "surgery"[All Fields]) OR "cardiac surgery"[All Fields] OR "cardiac surgical procedures"[MeSH Terms] OR ("cardiac"[All Fields] AND "surgical"[All Fields] AND "procedures"[All Fields]) OR "cardiac surgical procedures"[All Fields])) OR ("previous"[All Fields] AND ("thoracic surgery"[MeSH Terms] OR ("thoracic"[All Fields] AND "surgery"[All Fields]) OR "thoracic surgery"[All Fields] OR ("cardiac"[All Fields] AND "surgery"[All Fields]) OR "cardiac surgery"[All Fields] OR "cardiac surgical procedures"[MeSH Terms] OR ("cardiac"[All Fields] AND "surgical"[All Fields] AND "procedures"[All Fields]) OR "cardiac surgical procedures"[All Fields])) OR ("redo"[All Fields] AND ("surgery"[MeSH Subheading] OR "surgery"[All Fields] OR "surgical procedures, operative"[MeSH Terms] OR ("surgical"[All Fields] AND "procedures"[All Fields] AND "operative"[All Fields]) OR "operative surgical procedures"[All Fields] OR "general surgery"[MeSH Terms] OR ("general"[All Fields] AND "surgery"[All Fields]) OR "general surgery"[All Fields] OR "surgery s"[All Fields] OR "surgerys"[All Fields] OR "surgeries"[All Fields])) OR (("thoracic surgery"[MeSH Terms] OR ("thoracic"[All Fields] AND "surgery"[All Fields]) OR "thoracic surgery"[All Fields] OR ("cardiac"[All Fields] AND "surgery"[All Fields]) OR "cardiac surgery"[All Fields] OR "cardiac surgical procedures"[MeSH Terms] OR ("cardiac"[All Fields] AND "surgical"[All Fields] AND "procedures"[All Fields]) OR "cardiac surgical procedures"[All Fields]) AND ("history"[MeSH Terms] OR "history"[All Fields] OR "histories"[All Fields] OR "history"[MeSH Subheading])) OR ("cardiac surgical procedures"[MeSH Terms] OR ("cardiac"[All Fields] AND "surgical"[All Fields] AND "procedures"[All Fields]) OR "cardiac surgical procedures"[All Fields] OR ("cardiac"[All Fields] AND "surgical"[All Fields] AND "procedure"[All Fields]) OR "cardiac surgical procedure"[All Fields])) |
| --- | --- |
| **Google Scholar** | ("Type A acute aortic dissection") AND (previous cardiac surgery) |
| **Cochrane Library** | (Type A acute aortic dissection OR Type A aortic dissection) AND (previous cardiac surgery OR prior cardiac surgery) |

**Supplementary table 2.** Newcastle-Ottawa quality assessment scale for cohort studies

|  | Study name | | | | | | | | |
| --- | --- | --- | --- | --- | --- | --- | --- | --- | --- |
|  | Bjurbom et al. 2023 | Brown et al. 2022 | D’onofrio et al. 2023 | Estrera et al. 2010 | Ge et al. 2015 | Klodell et al. 2012 | Krebs et al. 2019 | Modi et al. 2011 | Rylski et al. 2014 |
| **Selection (4)** |  |  |  |  |  |  |  |  |  |
| Representativeness of the exposed cohort | * | * | * | * | * | * | * | * | * |
| Selection of the non-exposed cohort | * | * | * | * | * | * | * | * | * |
| Ascertainment of exposure | * | * | * | * | * | * | * | * | * |
| Demonstration that outcome of interest was not present at start of study | * | * | * | * |  | * | * | * |  |
| **Comparability (2)** |  |  |  |  |  |  |  |  |  |
| Comparability of cohorts based on the design or analysis | ** | ** | ** | * | ** | * | ** | * | ** |
| **Outcome (3)** |  |  |  |  |  |  |  |  |  |
| Assessment of outcome | * | * | * | * | * | * | * | * | * |
| Was follow-up long enough for outcomes to occur | * | * | * | * | * | * | * | * | * |
| Adequacy of follow up of cohorts | * | * | * | * | * | * | * | * | * |
| **Total (9)** | **9** | **9** | **9** | **8** | **8** | **8** | **9** | **8** | **8** |


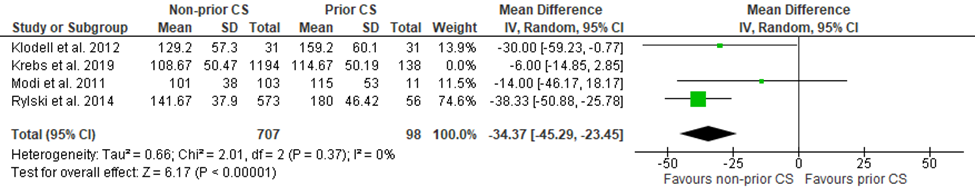

 **Supplementary figure 2 (S2).** Leave-one-out analysis of cross-clamp time **
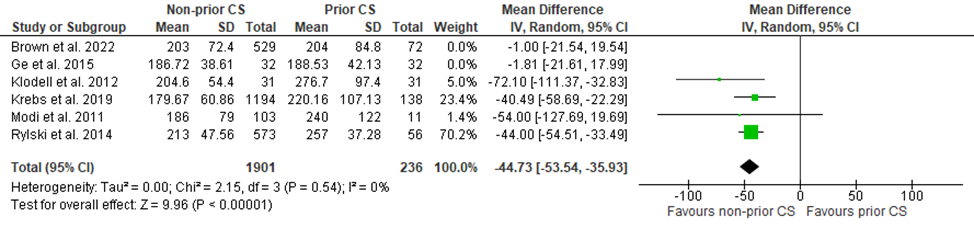
**

**Supplementary figure 3 (S3).** Removing outlier studies in the analysis of CPB time
